# Novel inflammatory markers in intracerebral hemorrhage: Results from Olink proteomics analysis

**DOI:** 10.1096/fj.202402183RR

**Published:** 2025-01-24

**Authors:** Ziliang Hu, Siqi Chen, Enhao Zhang, Liangzhe Wei, Jieyi Wang, Qing Shang, Xiang Gao, Yi Huang

**Affiliations:** ^1^ Cixi Biomedical Research Institute Wenzhou Medical University Ningbo Zhejiang China; ^2^ Department of Neurosurgery, Ningbo Key Laboratory of Nervous System and Brain Function The First Affiliated Hospital of Ningbo University Ningbo Zhejiang China; ^3^ Key Laboratory of Precision Medicine for Atherosclerotic Diseases of Zhejiang Province Ningbo Zhejiang China; ^4^ Department of Clinical Laboratory The First Affiliated Hospital of Ningbo University Ningbo Zhejiang China; ^5^ Department of Neurology The First Affiliated Hospital of Ningbo University Ningbo Zhejiang China

**Keywords:** biomarker, inflammation, intracerebral hemorrhage, Olink, proteomics

## Abstract

Inflammation is a crucial factor in intracerebral hemorrhage (ICH) pathophysiology, but specific inflammatory biomarkers in ICH patients remain unclear. This study aimed to identify novel circulating inflammatory biomarkers for improved ICH prediction and diagnosis. We profiled expression levels of 92 cardiovascular disease related proteins in plasma from 26 matched ICH patients and controls using Olink technology. Differentially expressed proteins were validated using ELISA and RT‐qPCR in a second matched cohort. Receiver operating characteristic (ROC) curves evaluated how well the diagnostic tests performed. The study identified 18 inflammatory‐related proteins with significantly different expression levels between ICH patients and controls. These proteins participate in critical biological processes and pathways, such as the regulation of inflammatory mediator secretion, cell death, immune cell proliferation and differentiation, pathogen response, and PI3K‐Akt and JAK–STAT pathways. Notably, we discovered for the first time that Kidney Injury Molecule‐1 (KIM1) is significantly upregulated in the plasma of ICH patients, suggesting its potential as a predictive and diagnostic biomarker for ICH. Validation results from ELISA and RT‐qPCR showed that Interleukin‐6 (IL‐6), Pentraxin 3 (PTX3), KIM1, and Galectin‐9 (Gal‐9) concentrations were markedly increased in the blood plasma and white matter of individuals with ICH. ROC analysis showed that the combined marker of IL‐6, PTX3, KIM1 and Gal‐9 had a high diagnostic efficacy (AUC = 0.941). This study identified a novel biomarker panel (IL‐6, PTX3, KIM1, Gal‐9) for ICH diagnosis. KIM1 upregulation in ICH patients is a novel finding, further investigation is needed into its expression and function in ICH.

## Graphical abstract

This study was divided into two phases. In the first discovery phase, 26 pairs of sex‐ and age‐matched case and control samples were collected, and the differentially expressed inflammation‐related factors were screened using Olink protein detection technology. In the second verification phase, another 26 pairs of sex‐ and age‐matched case and control samples were collected, ELISA and RT‐qPCR experiments were performed to verify the positive factors obtained in the first step.

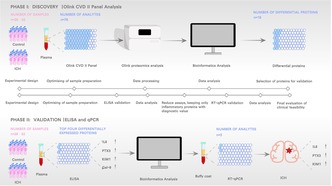

## 1 INTRODUCTION

Intracerebral hemorrhage (ICH) makes up 27.9% of stroke cases and is among the most devastating events, often leading to high rates of death and illness.^1^ In developed nations, improved blood pressure management has reduced the incidence of hypertensive ICH.^2^ However, the impact of ICH remains significant in developing countries despite these improvements.^3^ Brain injury following ICH includes primary damage caused by the physical trauma and blood infiltration into the brain parenchyma forming a hematoma, which compresses surrounding tissue, as well as secondary brain damage like brain edema, blood–brain barrier disruption (BBB), inflammation, and impairment of neuronal conduction.^4^

During brain damage after ICH, the body's defense response heavily relies on inflammation.^5^ This involves the activation and polarization of neuroglial cells, such as microglia or macrophages, towards the M1 phenotype, infiltration of peripheral inflammatory cells, and the release of pro‐inflammatory factors, which typically takes 1–3 days. Activation of microglia/macrophages leads to polarization, followed by the release of inflammatory mediators such as interleukin 1β (IL‐1β), tumor necrosis factor α (TNF‐α), and others. These events lead to neuronal apoptosis and BBB disruption, contributing to severe brain damage.^6^, ^7^, ^8^, ^9^

The detection of protein biomarkers plays a crucial role in disease prediction, screening, therapy, and long‐term outcome assessment. However, the use of advanced detection technologies generates a large amount of data, which also increases the complexity of analysis.^10^ Recently, research has shifted towards Olink proteomics to uncover the mechanisms of various injuries, including those involving inflammatory factors, following ICH. A previous study found that this technology exhibits excellent stability and reproducibility in detecting proteins in plasma samples from patients.^11^ Existing studies have identified numerous plasma predictors for ICH, such as IL‐6, PTX3, glucose, Factor XIII, and international normalized ratio (INR), which are used for ICH diagnosis and risk prognosis assessment.^12^, ^13^, ^14^ To explore new potential diagnostic biomarkers for ICH, this study aims to utilize the Olink CVD panel to screen for clinically relevant inflammation‐related proteins, followed by validation through ELISA and qPCR. The goal is to evaluate these proteins as diagnostic tools for ICH and to explore their application in current diagnostic practices. Given the complexity of ICH and the existing body of literature on diagnostic biomarkers, this study also seeks to complement current diagnostic methods by identifying new, clinically relevant biomarkers. While many diagnostic biomarkers for ICH have been proposed,^13^, ^15^ there remains a need for markers that can enhance early diagnosis and treatment stratification, especially in resource‐limited settings. In this study, our goal is to identify potential diagnostic biomarkers by detecting the differential expression of disease‐related proteins in the peripheral blood of ICH patients compared to a control group. We aim to evaluate the diagnostic value of these biomarkers for ICH, thereby complementing existing diagnostic methods and identifying new biomarkers for ICH.

## 2 MATERIALS AND METHODOLOGIES

### 2.1 Collection of samples (patients and study methodology)

**FIGURE 1:**
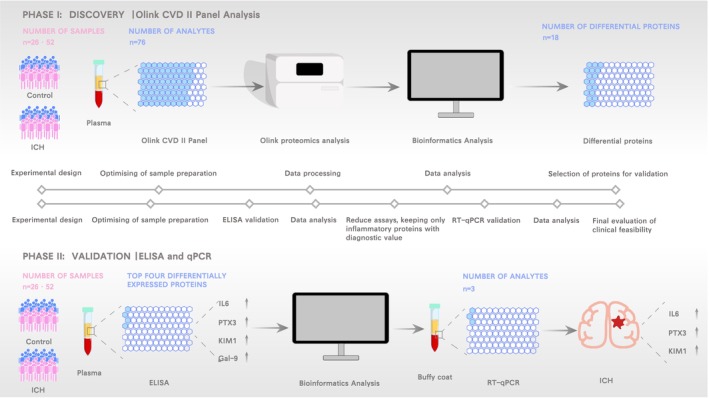
Flowchart of this study: The top represents the first stage, which is untargeted screening, and the bottom represents the second stage, which involves validating the screening results.

As shown in Figure 1, our study comprised two stages. In the first discovery phase, 26 pairs of gender‐ and age‐matched case and control samples were collected, and Olink protein detection technology was used to screen differentially expressed inflammatory‐related factors. In the second validation phase, another 26 pairs of gender‐ and age‐matched case and control samples were collected to perform enzyme‐linked immunosorbent assay (ELISA) and real‐time quantitative polymerase chain reaction (RT‐qPCR) experiments to verify the positive factors obtained in the first step. All case patients were inpatients with ICH in the Department of Neurosurgery of the First Affiliated Hospital of Ningbo University, and all control groups were patients with trigeminal neuralgia from the same department who were diagnosed to exclude any cerebrovascular disease. The subjects of this study are adult patients over 18 years of age, with inclusion criteria being ICH confirmed through head computed tomography (CT) or magnetic resonance imaging (MRI). Exclusion criteria are: (1) missing or incomplete key clinical data, such as patients who did not undergo a CT scan within 72 h post‐ICH; (2) presence of infections such as pulmonary or intracranial infections around the onset of ICH, such as bronchitis, lung infection, and intracranial infection; (3) severe mental or cognitive impairments prior to ICH that precluded examination; (4) the patients were examined for related diseases such as cerebral edema, brain abscess, encephalitis, or brain tumors.^16^ The diagnosis was independently verified by a minimum of two neurosurgeons. Prior to commencement, this study employed a detailed information disclosure process to ensure all participating patients fully understood the objectives, procedures, and potential risks of the research. Building on this foundation, all patients participating in this study were informed about the research process, signed informed consent forms, and approved the ethics application of the First Affiliated Hospital of Ningbo University (Ethics Approval No: 2024113A).

### 2.2 Plasma extraction and biochemical analyses

In the clinical sample collection process of this study, we drew approximately 6 milliliters of blood from patients within 6 h of admission. After centrifuging at 3200 rpm for 15 min at 4°C, the blood was fractionated, and the upper plasma layer and the middle buffy coat layer were separately extracted.^17^

The levels of glucose, total cholesterol (TC), triglycerides (TG), high‐density lipoprotein (HDL), low‐density lipoprotein (LDL), Creatinine (Cr), Blood Urea Nitrogen (BUN), Uric Acid (UA), and electrolytes (such as Sodium Na, Potassium K, Chloride Cl) were quantified using an automatic biochemical analyzer (Olympus AU2700, Tokyo, Japan). Apolipoprotein A (ApoA), apolipoprotein B (ApoB), and apolipoprotein E (ApoE) concentrations were measured using turbidimetric immunoassay techniques.

### 2.3 Neuroimaging and clinical outcomes

CT scans were collected within 24 h of hospital admission. The scan results were sent to the radiology department and analyzed by two neurosurgeons, who were blinded to the treatment task, in a random order. The patients' prognostic functional recovery was assessed based on the 90‐day functional disability level using the Modified Rankin Scale (mRS), which reflects the functional status and quality of life by evaluating the patient's ability to perform daily activities. The functional outcomes were assessed based on the mRS score range (using shift analysis). The mRS was evaluated through face‐to‐face follow‐up visits or structured phone interviews.

### 2.4 Proteomic analysis

In accordance with the manufacturer's guidelines, plasma samples from 26 ICH patients and 26 control patients were analyzed using the Olink® Target 92 CVD II panel, which is based on Proximity Extension Assay technology and has been extensively documented.^18^ Essentially, this technology involves highly specific binding of target proteins to antibody probes labeled with dual oligonucleotides, followed by quantitative detection of the resulting DNA sequences using microfluidic RT‐qPCR amplification of the oligonucleotide sequences. The final detection readings are shown as standardized protein expression values, which are log2 transformed. The Olink CVD panel was selected for its focus on inflammation‐related proteins, aligning with the study's goal of identifying diagnostic biomarkers for intracerebral hemorrhage (ICH). While the panel includes fewer than 100 proteins, its targeted approach allows for a focused exploration of clinically relevant biomarkers in this initial phase of research. Future studies may incorporate more comprehensive, unbiased methods, such as the Olink NGS panel, to further investigate the underlying mechanisms of ICH and validate our current findings.

### 2.5 Bioinformatics analysis

When analyzing the differential expression protein (DEP) sets between two groups, we used the R package “Olink® Analyze” and set a protein *p*‐value threshold of less than .05 to determine significant differential expression. We employed the ggplot2 package in R for data visualization and analysis, generating heatmaps and volcano plots to visualize the data.^19^ Additionally, we employed ggplot2, a versatile data visualization package in R, for conducting Gene Ontology (GO) enrichment analyses and for performing Kyoto Encyclopedia of Genes and Genomes (KEGG) pathway enrichment analyses. These analyses aimed to identify GO terms and KEGG pathways significantly enriched in proteins that showed differential expression. Additionally, to investigate the relationship in protein expression patterns between the two groups, a Spearman correlation analysis was conducted. We constructed protein–protein interaction (PPI) networks for differentially expressed proteins. Subsequently, we visualized these networks using igraph [1.4.1] and ggraph [2.1.0]. Finally, we used the ROCR package to generate ROC curves, which combined two or more indicators using Logistic regression analysis to display the combined diagnostic Area Under Curve (AUC) value in the bottom right corner legend, assessing the classification performance of the data.

### 2.6 ELISA analysis

To validate the precision of the positive factors discovered in the first stage, we newly collected 26 pairs of gender‐ and age‐matched case–control samples and used ELISA to detect the top four inflammatory factors with the most significant differences. The ELISA kits for the first four inflammatory factors are: Human Kidney Injury Molecule 1 (KIM1) (E‐EL‐H6029, Elabscience, Wuhan, China), Human Interleukin 6 (IL6) (E‐EL‐H6156, Elabscience, Wuhan, China), Human pentraxin3 (PTX3) (E‐EL‐H6081, Elabscience, Wuhan, China), and Human Galection‐9 (Gal‐9) (KE00175, Proteintech, Rosemont, USA). The assays were conducted and analyzed according to the manufacturers' protocols. A multimode microplate reader (Molecular Devices, California, USA) was used to measure absorbance at 450 nanometers for each well.

### 2.7 RT‐qPCR analysis

Based on the results from ELISA, we selected the top three inflammation proteins with the highest predictive value (IL6, PTX3, and KIM1) for validation at the mRNA level using RT‐qPCR. Total RNA was isolated from buffy coat samples using TRIzol reagent (Invitrogen, Carlsbad, California, USA). Subsequently, cDNA was synthesized from the RNA templates using the High‐Capacity cDNA Reverse Transcription Kit (TransGen Biotech, Beijing, China). With SYBR Green SuperMix from TransGen Biotech (Beijing, China), RT‐qPCR was conducted on a Roche LightCycler 480 system (Mannheim, Germany). Primers for RT‐qPCR were created with Primer6 software (Premier Biosoft, California, USA). The primer sequences are listed below: ACTB forward primer: 5′‐ATTGCCGACAGGATGCAGA‐3′, reverse primer: 5′‐CAGGAGGAGCAATGATCTTGAT‐3′, IL6 forward primer: 5′‐TAGAGTACCTCCAGAACAGATT‐3′, reverse primer: 5′‐AATAGTGTCCTAACGCTCATAC‐3′, PTX3 forward primer: 5′‐GCATAATAGGAACACTTGAGAC‐3′, reverse primer: 5′‐CTGACAGAGACACAGCATT‐3′, KIM1 forward primer: 5′‐GAATCTATGCTGGAGTCTGT‐3′, reverse primer: 5′‐GTCTGATGTGCTGATGTCT‐3′. The 2^−△△Ct^ method was used to determine mRNA expression levels.

### 2.8 Data analysis

Data analysis was performed using GraphPad Prism (v. 9.0, CA, USA) and SPSS (v. 25.0, Chicago, Illinois, USA). The mean substitution method was applied to handle missing data. ROC curves were constructed to evaluate the predictive accuracy of inflammatory proteins for ICH risk, with data significance defined at *p* < .05.

## 3 RESULTS

### 3.1 Clinical characteristics of participants

The clinical characteristics of the volunteers who participated in the Olink evaluation and validation cohorts are shown in Table 1. Blood glucose levels exhibited a notable rise among ICH patients compared to the control group in the Olink cohort (*p* = .018) and the validation cohort (*p* = .021). Moreover, there were no statistical disparities in age, sex distribution, TG, TC, HDL, LDL, ApoA‐I, ApoB, and ApoE between the case and control groups in both cohorts (*p* > .05, Table 1).

**TABLE 1:** Clinical characteristics of all participants.

Character	ICH (*n* = 26)	Control (*n* = 26)	*p* value
Phase I			
Age (year)	53.04 ± 10.79	53.08 ± 6.67	.988
Man (*n*)	13	13	.990
TG (mmol/L)	1.37 ± 0.73	1.59 ± 1.03	.373
TC (mmol/L)	4.51 ± 0.99	4.84 ± 0.81	.197
HDL (mmol/L)	1.14 ± 0.27	1.23 ± 0.25	.239
LDL (mmol/L)	3.01 ± 0.82	2.98 ± 0.62	.881
ApoA‐I (mg/dL)	1.19 ± 0.28	1.30 ± 0.22	.117
ApoB (mg/dL)	0.93 ± 0.26	0.90 ± 0.19	.588
ApoE (mg/L)	45.38 ± 9.75	49.58 ± 20.93	.384
Glucose (mmol/L)	6.22 ± 2.76	4.84 ± 0.86	.**018**
Phase II			
Age (year)	54.77 ± 12.92	54.50 ± 9.94	.933
Man (*n*)	13	13	.990
TG (mmol/L)	1.43 ± 0.69	1.32 ± 0.69	.555
TC (mmol/L)	4.49 ± 0.97	4.54 ± 0.71	.820
HDL (mmol/L)	1.19 ± 0.22	1.22 ± 0.29	.703
LDL (mmol/L)	2.82 ± 0.75	2.79 ± 0.53	.853
ApoA‐I (mg/dL)	1.21 ± 0.23	1.28 ± 0.25	.321
ApoB (mg/dL)	0.73 ± 0.21	0.71 ± 0.16	.665
ApoE (mg/L)	52.16 ± 20.06	46.21 ± 15.33	.241
Glucose (mmol/L)	5.47 ± 1.03	4.87 ± 0.71	.**021**

*Note*: Bold represents a statistical difference of less than .05.

Abbreviations: ApoA, apolipoprotein A; ApoB, apolipoprotein B; ApoE, apolipoprotein E; HDL, high‐density lipoprotein; LDL, low‐density lipoprotein; TC, total cholesterol; TG, triglycerides.

### 3.2 Analysis of inflammation‐related biomarkers by Olink

**FIGURE 2:**
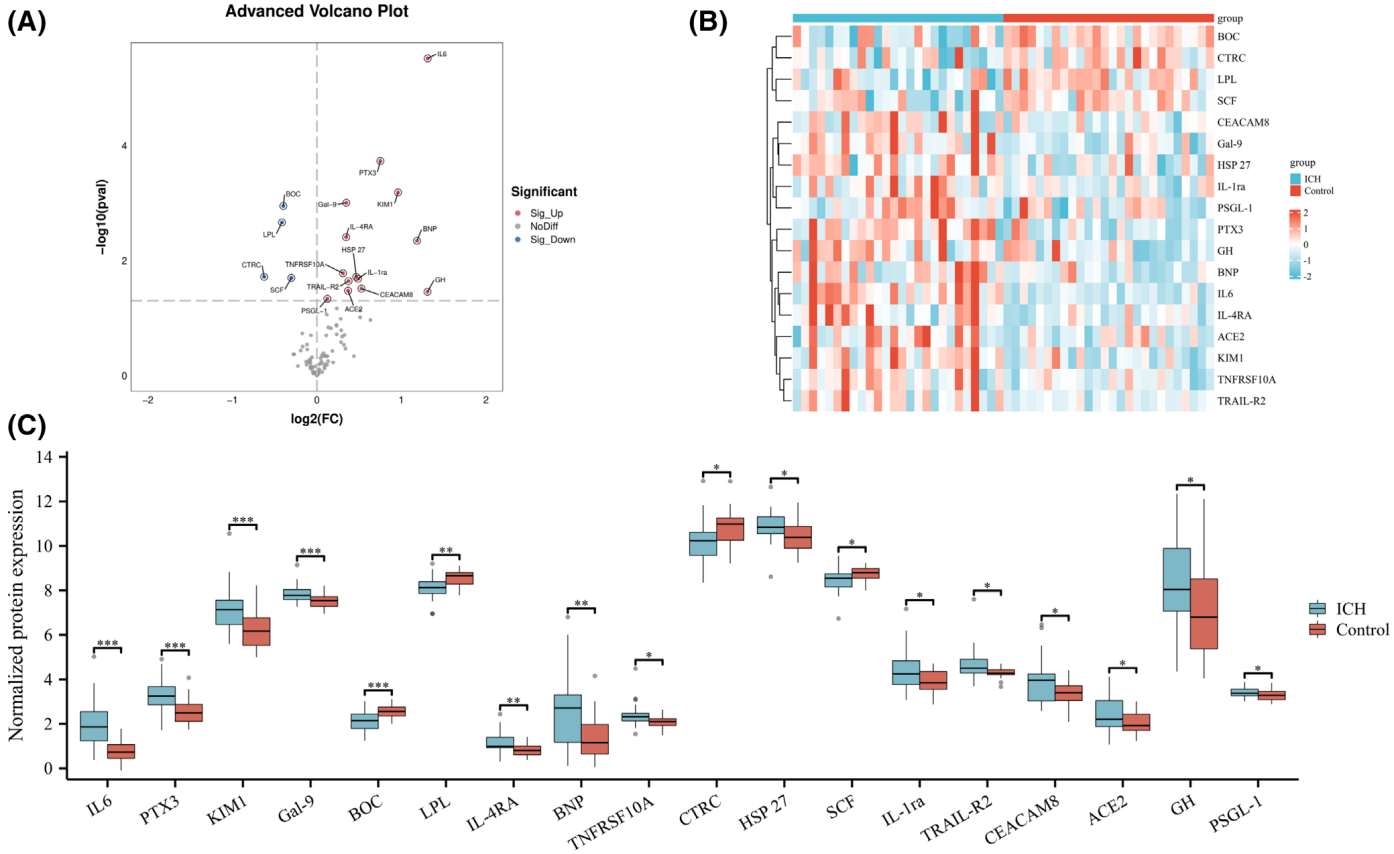
Differential expression of all inflammation‐related biomarkers between ICH and normal groups. (A) Volcano plot of 92 inflammation‐related biomarkers. (B) Heatmap of 18 differentially expressed proteins. (C) Boxplot of expression levels for 18 inflammation‐related biomarkers. Solid points outside the boxes represent observations 1.5 times beyond the interquartile range. *p < .05, **p < .01, ***p < .001.

According to the Olink CVD II panel analysis, we found 18 inflammation‐related proteins with differential expression between the two patient groups (Table 2). Fourteen proteins were upregulated in the ICH group, including IL‐6, PTX3, KIM1, Gal‐9, and Interleukin‐4 Receptor Alpha (IL‐4RA). Additionally, B‐type Natriuretic Peptide (BNP), TNF Receptor Superfamily Member 10A (TNFRSF10A), Heat Shock Protein 27 (HSP27), Interleukin‐1 Receptor Antagonist (IL‐1ra), TNF‐Related Apoptosis‐Inducing Ligand Receptor 2 (TRAIL‐R2), Carcinoembryonic Antigen‐Related Cell Adhesion Molecule 8 (CEACAM8), Angiotensin‐Converting Enzyme 2 (ACE2), Growth Hormone (GH), and P‐Selectin Glycoprotein Ligand‐1 (PSGL‐1) were upregulated. Conversely, four proteins were downregulated in the ICH group: Brother of CDO (BOC), Lipoprotein Lipase (LPL), Chymotrypsin‐C (CTRC), and Stem Cell Factor (SCF) (Figure 2A). Figure 2B shows a heatmap depicting the differential expression of these proteins between the two groups. The fold‐change differences in protein expression are further illustrated in the bar chart of Figure 2C.

**TABLE 2:** Significantly changed plasma inflammatory proteins between the ICH and control groups.

Assay	OlinkID	UniProt	Description	FC	*p* value
IL6	OID00390	P05231	Interleukin‐6	1.31	<.001
PTX3	OID00437	P26022	Pentraxin‐related protein PTX3	0.75	<.001
KIM1	OID00426	Q96D42	Hepatitis A virus cellular receptor 1	0.96	<.001
Gal‐9	OID00406	O00182	Galectin‐9	0.35	<.001
BOC	OID00386	Q9BWV1	Brother of CDO	−0.40	<.001
LPL	OID00446	P06858	Lipoprotein lipase	−0.41	<.001
IL‐4RA	OID00387	P24394	Interleukin‐4 receptor subunit alpha	0.35	<.001
BNP	OID00455	P16860	Natriuretic peptides B	1.18	<.001
TNFRSF10A	OID00391	O00220	Tumor necrosis factor receptor superfamily member 10A	0.31	.02
CTRC	OID00414	Q99895	Chymotrypsin‐C	−0.62	.02
HSP 27	OID00465	P04792	Heat shock protein beta‐1	0.47	.02
SCF	OID00408	P21583	Kit ligand	−0.30	.02
IL‐1ra	OID00389	P18510	Interleukin‐1 receptor antagonist protein	0.49	.02
TRAIL‐R2	OID00396	O14763	Tumor necrosis factor receptor superfamily member 10B	0.37	.02
CEACAM8	OID00436	P31997	Carcinoembryonic antigen‐related cell adhesion molecule 8	0.53	.03
ACE2	OID00457	Q9BYF1	Angiotensin‐converting enzyme 2	0.37	.03
GH	OID00417	P01241	Somatotropin	1.31	.03
PSGL‐1	OID00438	Q14242	P‐selectin glycoprotein ligand 1	0.12	.05

*Note*: Fold changes (FC) between ICH and control groups, calculated as log2. Student's *t*‐test was used to calculate *p*‐values (*p* < .05) indicating significantly different proteins.

### 3.3 Enrichment analysis and correlation analysis of differential inflammatory factors

**FIGURE 3:**
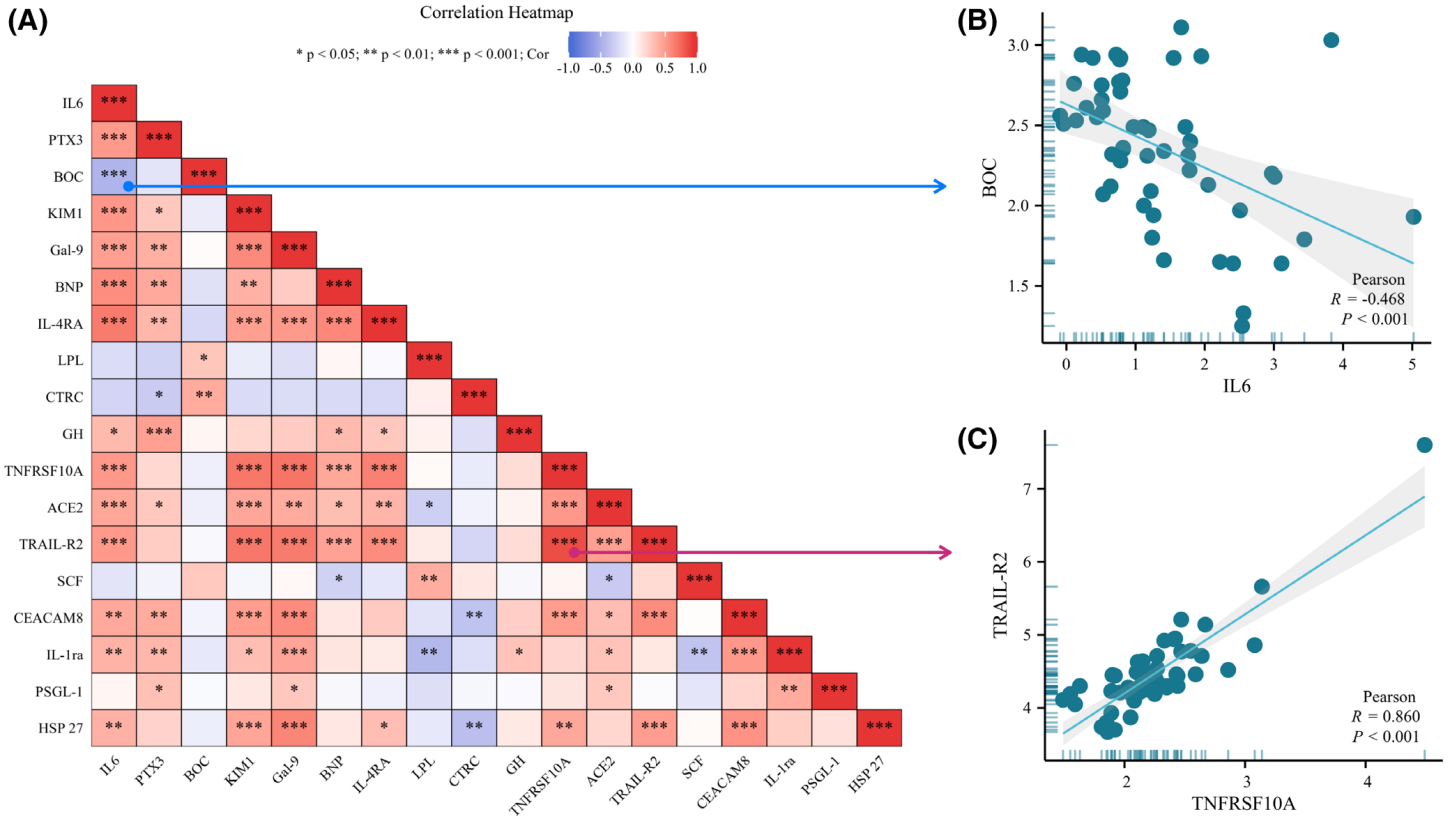
Correlation between differentially expressed inflammation‐related biomarkers in intracerebral hemorrhage (ICH) patients. (A) These scatter plots visualize the two pairs of inflammation‐related biomarkers with the highest correlation. (B) Brother of CDO (BOC) shows significant negative correlation with Interleukin 6(IL6). (C) Tumor necrosis factor‐related apoptosis‐inducing ligand receptor 2 (TRAIL‐R2) shows significant positive correlation with tumor necrosis factor receptor superfamily member 10A (TNFRSF10A).

Functional enrichment analysis was used to clarify the potential functions of differentially expressed proteins in plasma of ICH and control groups. The GO analysis revealed that the proteins exhibiting differential expression were primarily localized in the extracellular space, involved in binding of TNF‐related apoptosis‐inducing ligand (TRAIL) to its receptors, regulation of cell population proliferation, positive modulation of T helper 2 (Th2) cell differentiation, and acting as viral receptors (Figure S1A). The KEGG analysis results indicated that differentially expressed inflammatory factors were predominantly concentrated in the cytokines‐cytokine receptor interaction, hematopoietic cell lineage, measles, PI3K‐Akt and JAK–STAT signaling pathways (Figure S1B). We also evaluated the correlation between 18 inflammatory factors that exhibited significant differences between the case and control groups (Figure 3A). The results showed that BOC displayed the most notable inverse association with IL6 (Figure 3B), while TRAIL‐R2 had the most significant positive correlation with TNFRSF10A (Figure 3C). In addition, we also constructed a PPI network to measure the key roles of different inflammatory factors in ICH. The results showed that IL6 had the highest centrality score in the PPI network (Figure S2), suggesting their potential significance in the context of ICH.

### 3.4 Correlation between differential inflammatory factors and clinical characteristics

**FIGURE 4:**
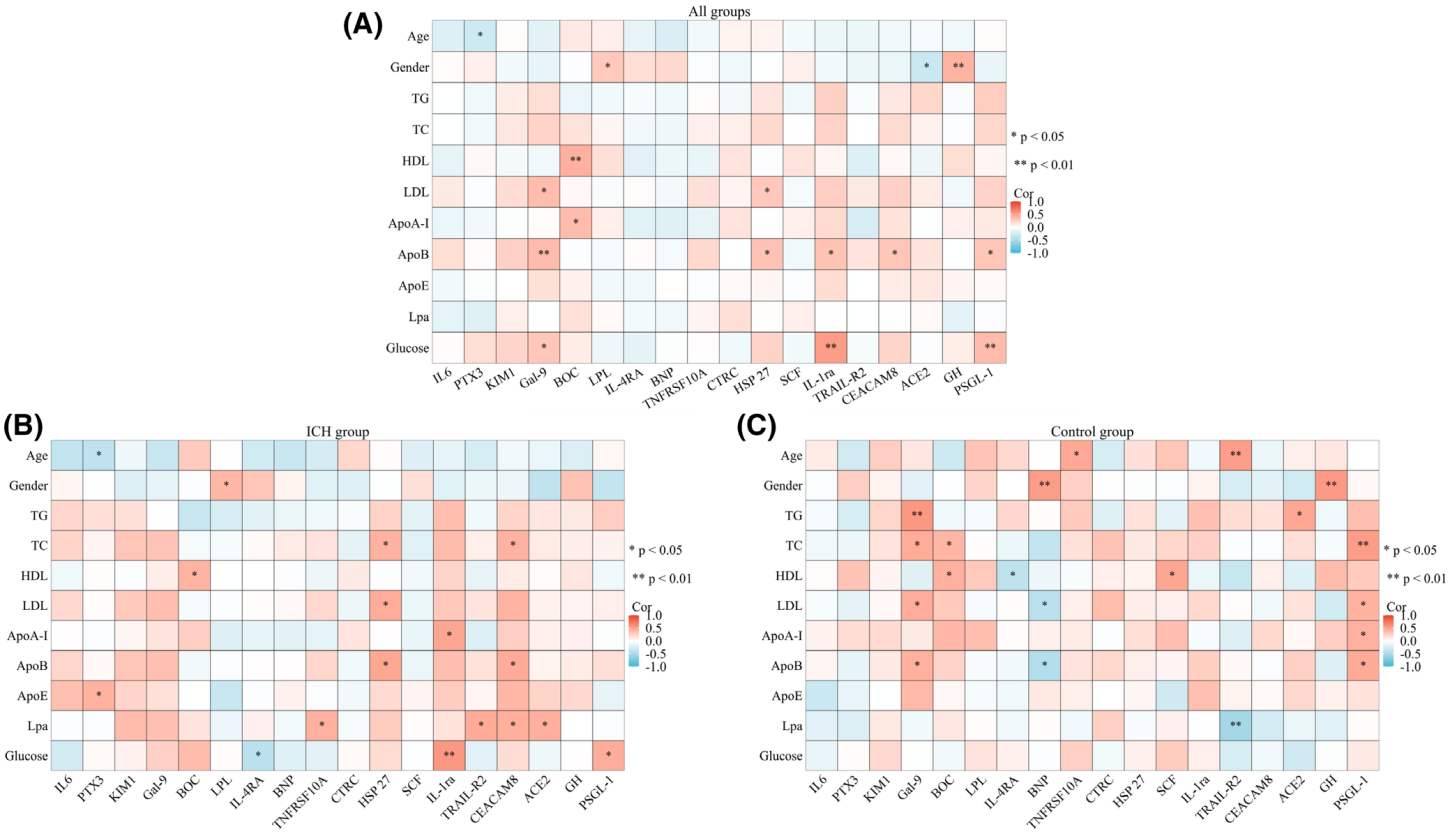
Heatmap of correlations between differentially expressed inflammation‐related biomarkers and clinical characteristics. (A) Heatmap showing correlations between 18 inflammation proteins and clinical features across all groups. (B) Heatmap displaying correlations between 18 inflammation proteins and clinical features in the ICH group. (C) Heatmap illustrating correlations between 18 inflammation proteins and clinical features in the control group. Red indicates positive correlation, blue indicates negative correlation, and white indicates no correlation. *p < .05, **p < .01.

We examined the association of 18 inflammation‐related proteins with the patient's clinical characteristics in each cohort. The findings indicated that age was negatively correlated with PTX3 in all participants (*p* < .05; Figure 4A). Gender exhibited a positive association with LPL (*p* < .05) and GH (*p* < .01) while showing an inverse relationship with ACE2 (*p* < .05; Figure 4A). It's noteworthy that PTX3 showed an inverse relationship with age and a positive association with ApoE in the ICH group (*p* < .05; Figure 4B), but there was no significant correlation in the control group (*p* > .05; Figure 4C). Similarly, Gal‐9 was closely correlated with TG (*p* < .01), TC, LDL, and ApoB in the control group (*p* < .05; Figure 4C), but there was no significant correlation in the ICH group (*p* > .05; Figure 4B).

### 3.5 ELISA validation

**FIGURE 5:**
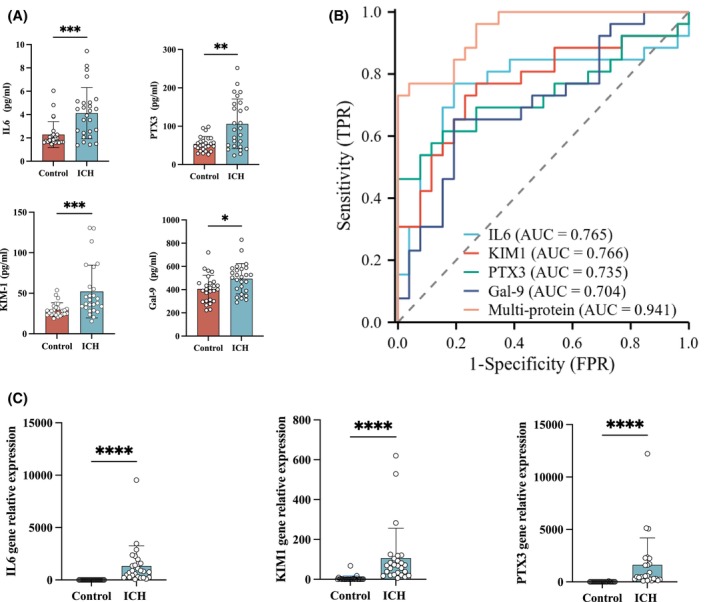
Validation of inflammatory cytokine levels in plasma and buffy coat of two patient groups. (A) ELISA validation of IL6, PTX3, KIM1, and Gal‐9 protein levels in plasma of ICH patients. (B) ROC curves of IL6, PTX3, KIM1, and Gal‐9 in ICH plasma. (C) RT‐qPCR validation of IL6, PTX3, and KIM1 levels in the buffy coat of ICH patients. *p < .05, **p < .01, ***p < .001, ****p < .0001.

To further validate the protein expression differences identified by Olink, we performed ELISA analysis on the four proteins exhibiting the most significant changes. The results indicated a notable increase in the plasma concentrations of these four proteins among ICH patients (IL‐6: *p* < .001; PTX3: *p* < .01; KIM1: *p* < .001; Gal‐9: *p* < .05, Figure 5A). Subsequently, ROC analysis was conducted to assess the diagnostic potential of each protein based on their plasma concentrations in ICH patients. IL‐6 demonstrated an Area Under the Curve (AUC) of 0.765 (95% CI: 0.621–0.909), with a Youden Index of 0.57692, sensitivity of 76.92%, and specificity of 80.77%. PTX3 yielded an AUC of 0.735 (0.591–0.879), with a Youden Index of 0.46154, sensitivity of 61.54%, and specificity of 84.62%. Notably, KIM1 exhibited the highest AUC of 0.766 (0.631–0.901) among the four proteins, suggesting its superior diagnostic value compared to the others. Youden Index for KIM1 was 0.5, with a sensitivity of 76.92% and specificity of 73.07%. Gal‐9 displayed an AUC of 0.704 (0.560–0.849), with a Youden Index of 0.46154, sensitivity of 65.39%, and specificity of 80.77%. Interestingly, combining all four proteins in a multi‐marker ROC analysis and a subsequent logistic regression model led to a markedly improved AUC of 0.941 (0.885–0.997) relative to KIM1 alone. This combined approach also yielded a superior Youden Index of 0.73007, with corresponding sensitivity and specificity rates of 76.93% and 96.15% (Figure 5B). These results underscore KIM1's potential as a promising diagnostic biomarker for ICH while also demonstrating the enhanced diagnostic efficacy achieved through a multi‐marker panel.

### 3.6 RT‐qPCR validation

The RT‐qPCR results corroborated those obtained from the ELISA experiments. In detail, IL‐6, PTX3 and KIM1 exhibited significantly elevated expression in the buffy coat of ICH patients compared to the control group (*p* < .0001, Figure 5C).

### 3.7 KIM1 and renal injury

**FIGURE 6:**
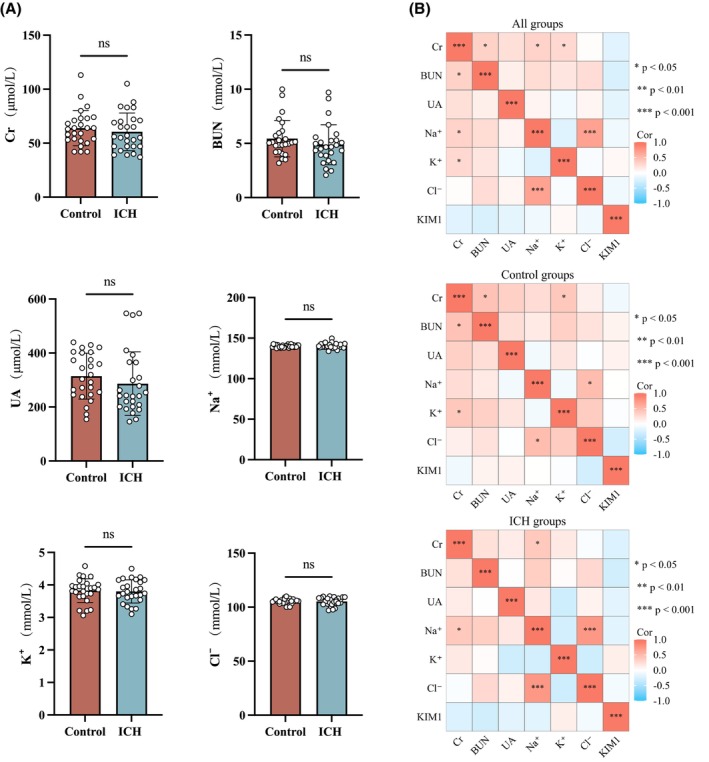
The renal function impairment in the two groups of patients and its correlation with KIM1. (A) Serum biochemical parameters of creatinine (Cr), blood Urea Nitrogen (BUN), uric acid (UA), and electrolytes in the two groups of patients. (B) Heatmap of the correlation between KIM1 and Cr, BUN, UA, and electrolytes. *p < .05, ***p < .001.

The blood biochemical results showed that there were no significant differences in renal function markers, including Cr, BUN, UA, sodium Na, potassium K, and chloride Cl, between the two groups (*p* > .05, Figure 6A). Additionally, KIM1 did not show a correlation with these indicators (*p* > .05, Figure 6B).

### 3.8 Correlation analysis of hemorrhage severity and prognostic indicators

**FIGURE 7:**
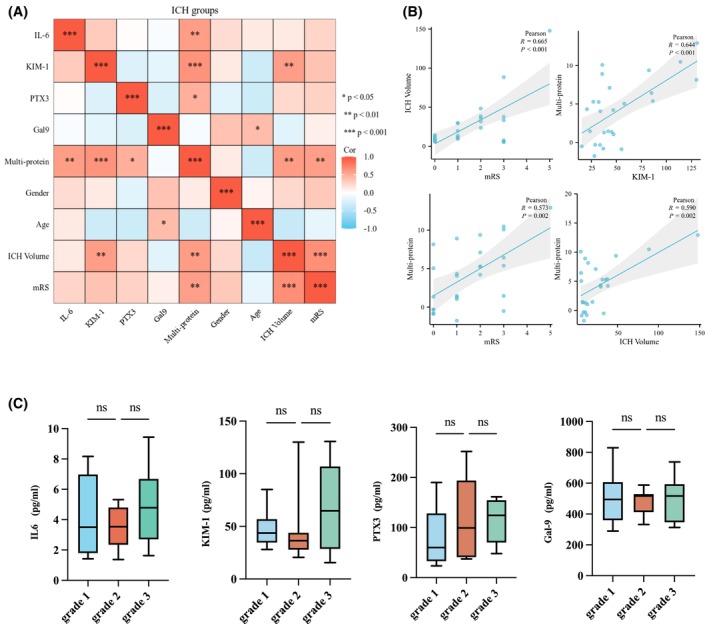
Correlation analysis of IL‐6, KIM‐1, PTX3, Gal‐9, and their combined index with ICH severity and prognosis in ICH patients. (A) Heatmap showing the correlations of IL‐6, KIM‐1, PTX3, Gal‐9, and their combined index with sex, age, hematoma volume, and the prognostic indicator mRS. (B) Scatter plots illustrating the correlations between ICH volume and mRS, the combined index and KIM‐1, the combined index and mRS, as well as the combined index and ICH volume. (C) Distribution of IL‐6, KIM‐1, PTX3, and Gal‐9 across different ICH grades. The line within the box represents the median, while the upper and lower edges of the box represent the first and third quartiles. *p < .05, **p < .01, ***p < .001.

We conducted a correlation analysis between ELISA results and the severity and prognosis of ICH patients. The results showed that ICH volume was positively correlated with poor recovery outcomes (*p* < .001; Figure 7A,B). Similarly, KIM‐1 levels were positively correlated with the combined index of four inflammatory markers (*p* < .001; Figure 7A,B). While individual proteins IL‐6, KIM‐1, PTX3, and Gal‐9 showed no significant correlation with mRS scores, their combined index was significantly positively correlated with mRS scores (*p* = .002; Figure 7A,B) and ICH volume (*p* = .002; Figure 7A,B). Figure 7C illustrates the expression levels of IL‐6, KIM‐1, PTX3, and Gal‐9 across different ICH grades (grades 1–3). While IL‐6, PTX3, and Gal‐9 levels appeared to increase with higher ICH grades, the differences did not reach statistical significance, potentially due to the limited sample size. These findings suggest a trend that warrants further investigation with a larger cohort (*p* > .05, Figure 7C).

## 4 DISCUSSION

This study explored inflammation protein biomarkers associated with ICH and conducted correlation analyses using blood samples from ICH patients and controls. The results showed: (1) Glycemic indicators were significantly elevated in ICH patients compared to controls in both study groups. (2) Eighteen proteins associated with inflammation were found to have differential expression between the ICH and control groups, enriched in various inflammation pathways, including regulation of inflammatory mediator secretion and action, mediation of cell death, regulation of immune cell proliferation and differentiation, response to pathogen infection, PI3K‐Akt, and JAK–STAT signaling pathway. (3) Inflammatory factors IL6, PTX3, KIM1, and Gal‐9 expression levels were markedly elevated in ICH cases compared to those in the control group. (4) The mRNA expression of IL6, PTX3, and KIM1 in the buffy coat of ICH patients was markedly elevated compared to those observed in individuals from the control group.

Our clinical data indicates that patients in the ICH group demonstrate markedly elevated blood glucose levels compared to those seen in the control group. Prior studies have suggested that acute cerebral hemorrhage patients often experience hyperglycemia due to increased secretion of adrenaline and glucocorticoids in response to stress, resulting in increased blood glucose levels. Moreover, elevated blood glucose in the acute phase of ICH is often linked with negative clinical consequences.^20^, ^21^ The specific mechanisms linking hyperglycemia to these outcomes are not fully understood, but it may involve inhibition of cellular junction proteins such as increased expression of matrix metalloproteinases (MMPs) and occludin, further disrupting the tight junctions in the BBB and reducing its permeability.^22^, ^23^, ^24^ Additionally, heightened blood glucose concentrations can contribute to increased production of reactive oxygen species (ROS) and initiation of inflammatory responses, causing oxidative damage and inflammation in brain tissue, exacerbating secondary injury following cerebral hemorrhage, and resulting in damage to neurons and vascular endothelial cells, further worsening brain tissue injury.^25^

Furthermore, we identified 18 proteins showing differential expression between the ICH and control groups, indicative of their involvement in inflammatory processes, which participate in various inflammatory pathways such as regulation of inflammatory mediators, PI3K‐Akt, and the JAK–STAT signaling pathway. These mechanisms can either improve or exacerbate the impact of inflammation on the disease. For instance, TRAIL‐R2 is a death receptor primarily inducing apoptosis by binding with TRAIL and also regulates inflammatory responses, influencing the release of inflammatory mediators and activation of inflammatory cells.^26^, ^27^ Additionally, research by Shengpan Chen and colleagues revealed that activation of the PI3K/Akt signaling pathway by TREM2 in bone marrow cells can mitigate neuroinflammation and neuronal apoptosis in an ICH mouse model,^28^ indicating that these inflammatory proteins may be involved in the pathogenesis of ICH through multiple mechanisms.

In this study, we measured the expression levels of IL6, PTX3, KIM1, and Gal‐9 and evaluated their efficacy as diagnostic biomarkers. The ROC curves for IL‐6, PTX3, and KIM1 demonstrated high diagnostic performance, with substantial AUC. However, Gal‐9 showed lower AUC values in distinguishing between healthy and diseased groups. Based on these results, we focused further validation and functional studies on IL‐6, PTX3, and KIM1. Confirmation of the association between IL‐6 levels and ICH was established. IL6 is a pro‐inflammatory cytokine widely studied as an indicator of systemic inflammation in acute brain injury.^29^, ^30^, ^31^, ^32^ Previous research has reported a correlation between IL6 levels and functional outcomes following ICH. Studies by Audrey C. Leasure and colleagues found that higher IL‐6 concentrations in ICH patients were associated with poorer functional outcomes and larger hemorrhage volumes.^13^ Our data indicate elevated IL‐6 levels in both plasma and the subarachnoid space of ICH patients. However, further data are needed to establish whether IL‐6 causally contributes to worse outcomes.

PTX3 is an acute‐phase reactant protein that plays a critical role in inflammatory processes and immune system modulation, rapidly produced in response to infection, injury, or inflammatory stimuli.^33^ Studies have found that elevated levels of PTX3 during the initial phase, they are linked to unfavorable results in patients with ICH, including higher mortality and disability rates.^14^ PTX3 levels hold potential as a biomarker for predicting outcomes in ICH. Our findings confirm this potential, but further research into the mechanisms and functions of PTX3 is needed to elucidate the pathological processes of ICH and provide potential targets for developing new therapeutic strategies.

We have identified a novel association between KIM1 and ICH. To our knowledge, KIM1 is an immunoglobulin superfamily protein typically increased in the proximal tubules of damaged or diseased kidneys.^34^, ^35^, ^36^, ^37^ Most studies on KIM1 focus on kidney injury, highlighting its high expression in renal tubular epithelial cells.^37^ However, research on KIM1 in the nervous system is relatively limited, possibly due to its lower expression levels in brain tissues and thus not widely explored. Despite its predominant expression in the kidneys, studies suggest its expression in certain neurological conditions such as brain death and traumatic brain injury.^38^, ^39^ Moreover, some emerging studies suggest a potential association between KIM1 and ICH. For instance, Xiaoping Cai et al. reported that serum amyloid A (SAA) is linked to vascular inflammation and dysfunction in cardiovascular and kidney diseases through its association with KIM1.^40^ This endothelial damage may play a critical role in blood–brain barrier disruption caused by ICH. Additionally, Nicolas S. Merle et al. found that Kim‐1 in renal tubules of mice is closely associated with tissue and vascular inflammation.^41^ Since systemic inflammatory response is a significant feature of secondary injury in ICH, this association supports the potential of KIM1 as a diagnostic biomarker for ICH. Our study reveals increased expression of KIM1 following ICH, suggesting its potential role in inflammatory responses and tissue repair following neural injury. Additionally, we excluded renal injury in both groups as much as possible, indicating that the KIM1 in the plasma is most likely derived from the nervous system. Further exploration is needed in the future, including cell culture and animal studies, to elucidate the molecular pathways and pathological mechanisms involved. The importance of this research is found in identifying KIM1 as a potential novel biomarker for predicting and diagnosing ICH.

Gal‐9 is a glycoprotein belonging to the galectin family, which has been proven to play a crucial regulatory role in inflammation and immune response.^42^, ^43^ Current investigations have demonstrated that Gal‐9 levels are elevated in a rat model of ICH, accompanied by an increase in M2 microglia and related anti‐inflammatory factors. This suggests that Gal‐9 is significant in the context of ICH, and its levels could serve as a potential biomarker for ICH prognosis.^44^ Our research confirmed this through peripheral blood testing, but the specific mechanisms of Gal‐9 in ICH remain unclear, requiring further research to explore the pathological processes of ICH.

Although this study proposes IL‐6, KIM‐1, PTX3, and Gal‐9 as potential diagnostic biomarkers for ICH, challenges in differential diagnosis remain in clinical practice. ICH shares similar clinical symptoms with other neurological conditions, such as subdural empyema, subdural hygroma, or meningioma, including headache, altered consciousness, and focal neurological deficits.^45^, ^46^, ^47^ However, the inflammatory mechanisms underlying these conditions differ, leading to distinct biomarker expression patterns among these diseases.

For instance, IL‐6 is a common inflammatory marker that is elevated in numerous inflammatory conditions.^48^ Relying solely on IL‐6 as a specific biomarker for ICH may have limitations, especially since IL‐6 elevation is also observed in other diseases such as infections or tumors.^49^ In contrast, KIM‐1, a marker associated with kidney injury, appears to show specificity in ICH patients, making it a more promising candidate for diagnosis. Moreover, PTX3 and Gal‐9 have been found to be closely associated with the inflammatory response in ICH.^14^, ^44^ Therefore, the combined use of these biomarkers could enhance the accuracy of differential diagnosis.

Our research results indicate that the combined indicators of IL‐6, KIM‐1, PTX3, and Gal‐9 not only serve as diagnostic biomarkers for ICH but also provide strong support for assessing the severity and prognosis of the disease. Notably, the unique potential of KIM‐1 in ICH prognosis evaluation warrants further investigation. Although this study found certain trends in the levels of these biomarkers across different ICH grades, statistical analysis did not show significant differences, possibly due to the small sample size. Therefore, future studies should consider expanding the sample size to improve statistical power and more accurately validate the clinical significance of these biomarkers. Additionally, longitudinal follow‐up studies could be conducted to verify the predictive capacity of these biomarkers and combine them with patient imaging data and other clinical parameters to develop a more comprehensive prognostic assessment model. This would help further understand the pathophysiological mechanisms of ICH and optimize clinical management for patients. Additionally, future studies could explore the expression profiles of these biomarkers in other neurological conditions, particularly in subdural empyema, subdural effusion, or meningioma, to confirm their practical clinical applicability.

Certainly, this study still has many limitations. Firstly, as mentioned earlier, due to the limited sample size, we were unable to collect enough samples, which may affect the statistical power. Secondly, while we have verified the association between KIM‐1 and ICH through preliminary experiments, further studies are needed to explore the specific pathways and interactions of KIM‐1 in ICH more thoroughly.

## 5 CONCLUSION

This study identified a novel biomarker panel (IL6, PTX3, KIM1, and Gal‐9) for ICH prediction and diagnosis. Notably, we report the first‐time observation of KIM1 upregulation in ICH patients. However, further investigation is needed to elucidate its specific expression patterns and functional role in ICH pathophysiology.

## AUTHOR CONTRIBUTIONS

Conceptualization: X.G. and Y.H.; methodology: Z.H., S.C., E.Z., L.W., J.W., Q.S.; writing—original draft preparation: Z.H.; writing—review and editing, Y.H. and X.G.; project administration: Y.H. All authors have read and agreed to the published version of the manuscript.

## FUNDING INFORMATION

This study was supported by grants from the Ningbo Top Medical and Health Research Program (2022020304) and the Ningbo Natural Science Foundation (2023J019 and 2022Z125).

## DISCLOSURES

The authors declare no conflict of interest.

## ETHICS APPROVAL AND CONSENT TO PARTICIPATE

Not applicable.

## CONSENT FOR PUBLICATION

Not applicable.

## Supporting information


Figure S1.

## Data Availability

Data are contained within the article.
