# Supplementary material for: Novel inflammatory markers in intracerebral hemorrhage: Results from Olink proteomics analysis
Source: FASEB J. 2025 Jan 24;39(2):e70341. doi: 10.1096/fj.202402183RR (PMC11760662; doi:10.1096/fj.202402183RR)
Supplement: Supplementary file 1 — Figure S1. [file FSB2-39-e70341-s001.docx]

_
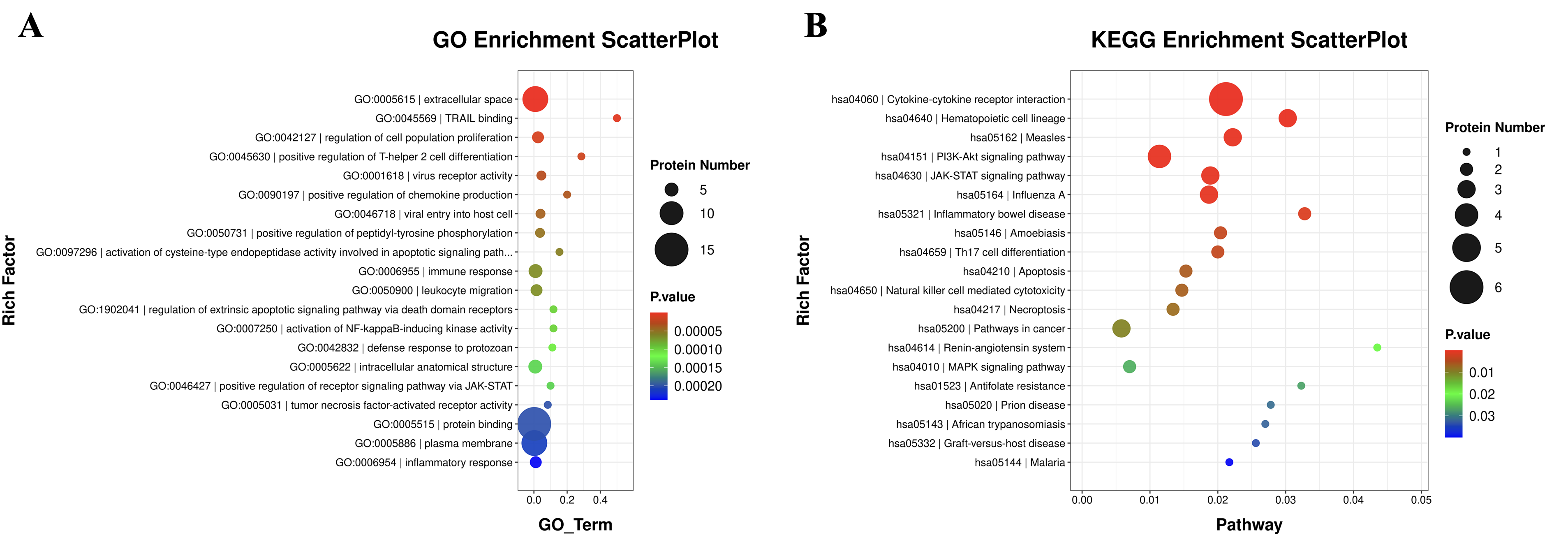
_

**Figure S1**: Enrichment analysis of differentially expressed inflammation-related proteins using Gene Ontology (GO) and Kyoto Encyclopedia of Genes and Genomes (KEGG). (A) Top 20 enriched GO terms based on background of all annotated proteins. (B) Top 20 enriched KEGG pathways based on background of all annotated proteins.

_
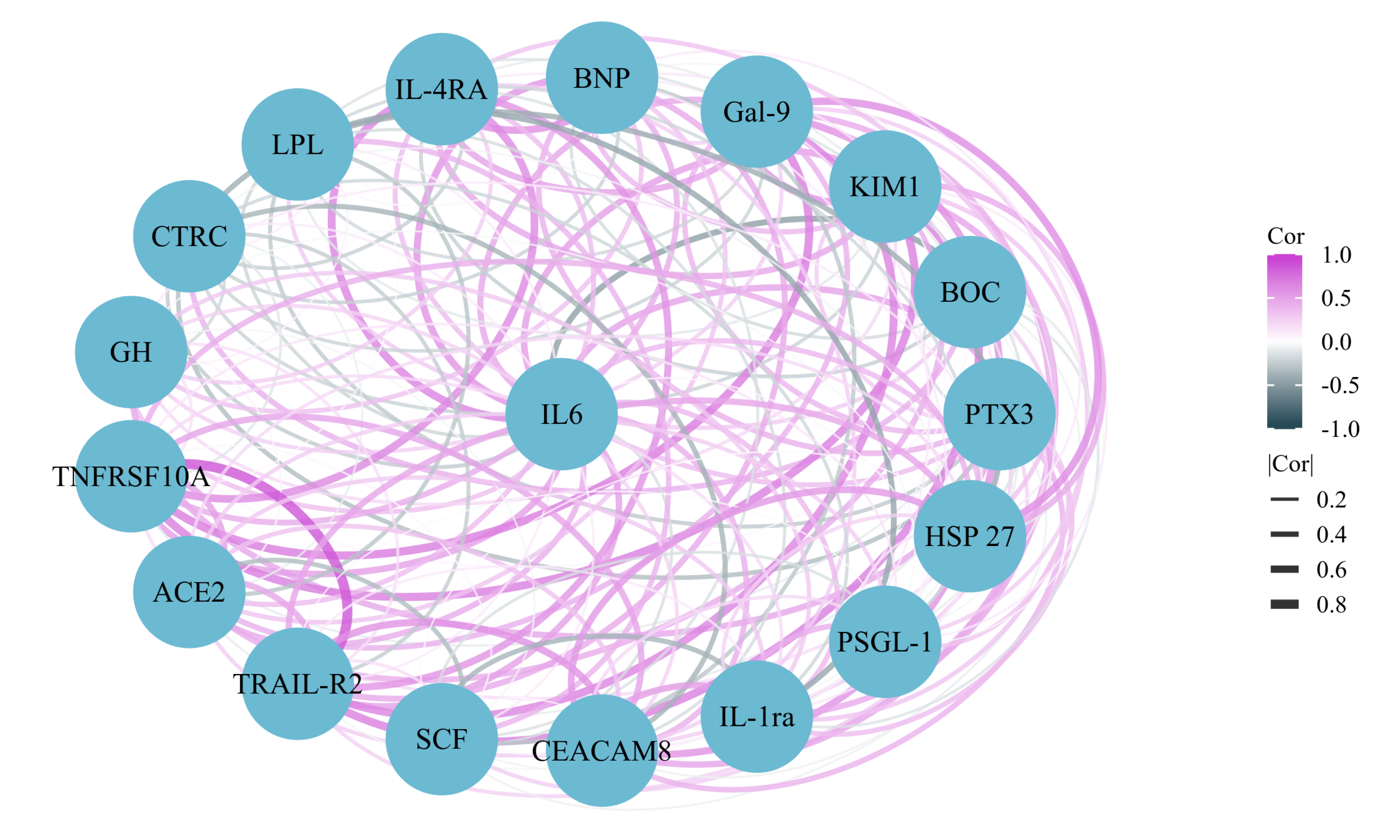
_

**Figure S2**: Protein-Protein Interaction (PPI) network analysis of inflammation-related differentially expressed proteins.
